# Rehabilitation Utilization following a Work-Related Traumatic Brain Injury: A Sex-Based Examination of Workers’ Compensation Claims in Victoria, Australia

**DOI:** 10.1371/journal.pone.0151462

**Published:** 2016-03-16

**Authors:** E. Niki Guerriero, Peter M. Smith, Mary Stergiou-Kita, Angela Colantonio

**Affiliations:** 1 Rehabilitation Sciences Institute, University of Toronto, Toronto, Ontario, Canada; 2 The Institute for Work & Health, Toronto, Ontario, Canada; 3 The Dalla Lana School of Public Health, University of Toronto, Toronto, Ontario, Canada; 4 School of Public Health and Preventive Medicine, Monash University, Melbourne, Australia; 5 Department of Occupational Science and Occupational Therapy, University of Toronto, Toronto, Ontario, Canada; 6 Toronto Rehabilitation Institute - University Health Network, Toronto, Ontario, Canada; University of Florida, UNITED STATES

## Abstract

**Objectives:**

To report on and examine differences in the use of four types of rehabilitation services (occupational therapy, physiotherapy, psychology, and speech therapy) by men and women following a work-related traumatic brain injury in Victoria, Australia; and to examine the importance of demographic, need, work-related and geographic factors in explaining these differences.

**Methods:**

A retrospective cohort design was used to analyze 1786 work-related traumatic brain injury workers’ compensation claims lodged between 2004 and 2012 in Victoria, Australia. ZINB regressions were conducted for each type of rehabilitation service to examine the relationship between sex and rehabilitation use. Covariates included demographic, need-related, work-related, and geographic factors.

**Results:**

Out of all claims (63% male, 37% female), 13% used occupational therapy, 23% used physiotherapy, 9% used psychology, and 2% used speech therapy at least once during the first year of service utilization. After controlling for demographic, need-related, work-related, and geographic factors, women were more likely to use physiotherapy compared to men. Men and women were equally likely to use occupational therapy and psychology services. The number of visits in the first year for each type of service did not differ between male and female users.

**Conclusions:**

Our findings support a sex-based approach to studying rehabilitation utilization in work-related populations. Future research is needed to examine other factors associated with rehabilitation utilization and to determine the implications of different rehabilitation utilization patterns on health and return-to-work outcomes.

## Introduction

Survivors of traumatic brain injury (TBI) often live with devastating consequences, including cognitive and physical impairments; functional disability; and disturbances in psychosocial functioning [1,2] that leads to long-term utilization of healthcare services and extreme healthcare costs. [3–5] In Victoria, Australia, there were 7 888 hospital admissions due to TBI between 2009–2010 and the lifetime costs per incidence of moderate and severe TBI cases were estimated to be $2.6 million and $5.0 million, respectively. [5] TBI occurring in the workplace or work-related TBI is among the most serious types of workplace injuries, as it poses large economic costs [2] and has been shown to have significantly longer recovery times compared to other work-related injuries. [6]

Although similar, the epidemiological factors and outcomes associated with work-related TBI are distinct from the overall TBI population [2]. For example, compared to non-work-related TBI, the work-related TBI population is significantly older at the time of injury; has a significantly larger proportion of men, and has different distributions of mechanisms of injury [1, 2]. Thus, since the characteristics of individuals within work-related and non-work-related TBI populations are different, it is important to study and build an understanding of work-related TBI separately from the rest of the population.

Fortunately, there is evidence that post-acute care and rehabilitation programs have significant benefits for survivors of TBI. [7] Rehabilitation programs aim to maximize a person’s functioning and participation based on their individual goals and needs. [8] In the literature, an interdisciplinary approach to rehabilitation is recommended for persons with a TBI to address the wide range of symptoms characteristic of this type of injury. [9] It is apparent that rehabilitation is important for survivors of TBI, however the nature of and the extent to which different rehabilitation services are being used and accessed by this population, particularly in a workers’ compensation environment, is not well understood.

In addition, research in this area has traditionally focused on men so we currently have a limited understanding of the similarities and differences between sexes both in general and within the context of rehabilitation utilization specifically. However, in the context of healthcare utilization, existing studies on other types of injuries have shown that men and women utilize healthcare services differently [10–13] with some providing reasons such as men and women having different health-seeking behaviours [14] and physicians referring male and female patients with the same injury to different services. [10] For example, in one study on patients with moderate knee osteoarthritis, the odds a male was referred to a total knee arthroplasty by a physician was 4.2 times the odds for a female. [10] Thus, it is plausible that men and women presenting with a TBI have different patterns of rehabilitation use post-injury.

In TBI populations that are not exclusively work-related, some research has looked at the patterns of healthcare and/or rehabilitation use overall [15–18] and by type of service [19–23] post-TBI. The latter studies have found that physicians, physiotherapists, occupational therapists, psychologists, and speech therapists are most commonly visited by persons with a TBI; and in one study that briefly looked at gender, men and women were equally likely to use different rehabilitation services. [23] However, most of these studies were not population-based, utilized subjective data (e.g., questionnaires), or did not include the entire range of TBI severity (e.g., used hospital data that may not have captured milder injuries that did not require hospitalization). Regarding work-related TBI populations specifically, very few studies have examined rehabilitation use; Wrona [24] described the number of work-related TBI claims that used rehabilitation services but did not examine different types of rehabilitation services specifically or examine differences between men and women, and Kristman et al. [25] described health service use among mild work-related TBI claimants and by sex, but did not present different types of rehabilitation services separately. There are unique characteristics and barriers/enablers to accessing rehabilitation that are specific to a workers’ compensation environment (e.g., rehabilitation referral processes, the existence of case managers, insurance eligibility, etc.), so it is important to look at rehabilitation use within the work-related TBI population separately from the overall TBI population in order to accurately inform the planning and management of rehabilitation of these injured workers.

To address these research gaps, the objectives of this study were to (i) report on and examine differences in the use of four types of rehabilitation services (occupational therapy, physiotherapy, psychology, and speech therapy) by men and women following a work-related TBI in Victoria, Australia; and ii) examine the importance of demographic, need, work-related, and geographic factors in explaining these differences. Based on previous research (not specifically related to work-related TBI) we hypothesized that men and women will differ in their use of rehabilitation services over the first year following a work-related TBI.

## Methods

### Study Design

A retrospective cohort design was used to analyze workers’ compensation claims lodged over a nine-year period between 2004 and 2012.

### Workers’ Compensation System and Data Source

WorkSafe Victoria (WSV) is a state government authority responsible for occupational health and safety legislation and providing compensation and rehabilitation to injured workers in the state of Victoria, Australia. [26] Approximately 85–90% of workers in Victoria are insured under WSV. Groups excluded include federal government employees, sole traders, and employees of self-insured companies [27] and WSV does not cover injuries occurring during travel to or from work. A claim may be lodged once a worker is off work for more than ten days *or* medical expenses have surpassed a certain threshold amount (e.g., $592 in 2010/2011). Employers are responsible for providing income replacement for the first 10 days a worker is unable to work and covering medical expenses up to the threshold amount; thereafter WSV covers all costs.

The current study analyzed data extracted from the Compensation Research Database (CRD) and provided by WSV. The CRD is maintained by the Institute for Safety, Compensation, and Recovery Research (ISCRR) at Monash University and contains population-wide case-level data of workers’ compensation claims collected from WSV. The CRD includes detailed information related to: each claimant and their injury (e.g., age, sex, occupation, industry, nature of injury, etc.); services received by each claimant (e.g., rehabilitation services, return-to-work programs, etc.); payments made to service providers by WSV; medical certificates issued to each claimant; and hospital admissions of each claimant. For the purposes of this study, de-identified workers’ compensation claims data were extracted from the CRD for the nine-year period 2004–2012.

### Study Population

The study population included male and female workers, aged ≥15 years, across all occupations and industries, who sustained a work-related TBI between January 1, 2004 and December 31, 2012 and survived for at least one year post-injury. Cases before January 1, 2004 were excluded because of significant changes in workers’ compensation legislation and coding of injuries in the years prior. For the purposes of this study, a work-related TBI was defined as an injury claim with the nature of injury coded as “intracranial injury” as per the Australian Standard Type of Occurrence Classification System [28] which is derived from the International Classification of Diseases, 10th revision, Australian Modification. According to the Australian Standard Type of Occurrence Classification System, intracranial injuries include cerebral contusion, cerebral laceration, traumatic extra-dural haemorrhage/haematoma, traumatic subarachnoid haemorrhage, traumatic sub-dural haemorrhage/haematoma, concussion, headache from blow to the head, and other unspecified intracranial injuries. When a claimant has multiple injuries, the most serious injury will always be documented in the claim file. Fortunately, intracranial injuries are considered the most serious type of injury above all other injuries and so the possibility of missing claims with a diagnoses of intracranial injury was not a major concern.

### Main Outcome: Rehabilitation Utilization

Rehabilitation utilization was examined for four different types of rehabilitation services: occupational therapy, physiotherapy, psychology, and speech therapy. These four rehabilitation types were chosen because, in previous studies examining other TBI populations (i.e., not exclusively work-related), these services are the most common rehabilitation services received [19–23]. For each type of service we defined measures of use (yes/no) and, for those who used each service, a count of the number of times each service was used. We examined service use over the first 365 days post-injury, not including days when the claimant was an inpatient in hospital (referred to as ‘one-year of service utilization’). The reason for not counting hospital days in our 365 days post-injury is because service use is captured unevenly during hospital stays (e.g. physiotherapy services used while an inpatient are not capture in the administrative database at WSV). This approach ensured that each claimant had an equal number of days of possible out-of-hospital service use.

### Main Independent Variable: Sex

Our primary independent variable of interest was ‘sex’, defined as male or female based on the claim file.

### Covariates

Organization of the covariates of interest was guided by the Andersen and Newman model of determinants of health care service utilization. [29] Using this model, we grouped our covariates into demographic characteristics, need factors, work-related factors, and geographic factors. Each of these groupings is outlined in further detail below.

Demographic characteristics outside of sex included ‘age’, defined as the age (years) of the claimant at the time of injury.

Need factors included ‘length of first hospitalization’, and ‘number of days compensated’. Length of first hospitalization was defined as the total number of days spent in hospital during a claimants’ first hospital admission, where this first hospital admission occurred within seven days of the injury. Forty-eight hours or less between two hospital episodes were considered to be one episode. This variable had six levels (not hospitalized, hospitalized for 0 days [day patient], 1 day, 2–3 days, 4–7 days, >7 days). Number of days compensated was defined as the total number of days the claimant was compensated with wage replacement by WSV from their first date of incapacity to 30 days post-incapacity. This variable had five levels (0 days, 1–5 days, 6–10 days, 11–15 days, 16–30 days).

Work-related factors included ‘occupational skill level’, ‘employment type’, and ‘employer remuneration size’. Occupational skill level was coded into levels 1–5 (Level 1 represents the highest skill level, Level 5 represents the lowest skill level) and is defined in terms of formal education and training; previous experience; and on-the-job training. [30] Employment type was categorized as full-time (working 35 hours or more per week), part-time (working less than 35 hours per week), or other. Employer remuneration size was defined as the employer’s remuneration and was categorized as small (less than $1 million), medium ($1–20 million), large (greater than $20 million), or government.

Geographic factors included ‘remoteness’ and ‘socioeconomic advantage/disadvantage’. Remoteness was categorized into “Major Cities of Australia”, “Inner Regional Australia”, “Outer Regional Australia”, and “Remote Australia” based on the Accessibility/Remoteness Index of Australia. [31] Socioeconomic advantage/disadvantage was coded based on each claimants residential location into deciles 1–10 (Decile 1 represents the most disadvantaged, Decile 10 represented the most advantaged) using the Index of Relative Socio-economic Advantage and Disadvantage derived from 21 census variables related to a geographic area’s advantage (high education, high income, etc.) and disadvantage (low education, low income, etc.). [32]

‘Injury year’ was used to represent all unmeasured structural changes across study years not captured by other environmental factors.

### Statistical Analysis

Data analysis was performed using SAS V.9.3.

An initial series of analyses examined frequencies and descriptive statistics for our study covariates and main outcome stratified by sex. Chi-square (Χ^2^) tests of independence were conducted to determine if men and women differed in any of these variables. Fisher’s exact test was used when cells had an expected count of <5. For all tests, p<0.05 was considered statistically significant and claimants with missing data were excluded listwise.

We then conducted a series of Zero-Inflated Negative Binomial (ZINB) models separately for each type of rehabilitation service. The primary goal behind these analyses was to examine the differences between men and women in their use of rehabilitation services, and to separately examine the relative importance of each grouping of covariates on this relationship. A ZINB model is a modified Poisson model used for data with excess zeros and overdispersion, and includes two components: (i) a logistic regression component modeling the probability of an event occurring out of two possible outcomes (i.e., in our study, the probability of using (versus not using) a particular type of rehab) and (ii) a negative binomial regression component modeling the expected count (i.e., in our study, the expected visit count for a particular type of rehabilitation service).

For each type of service (excluding speech therapy), five ZINB models were tested: (1) a simple model with age and injury year as the only covariates; (2) a model additionally adjusting for need factors; (3) a model additionally adjusting model 1 for work-related factors; (4) a model additionally adjusting model 1 for geographic factors; and (5) a final model adjusting for all factors together. As stated above, the primary interest in each of the models was the effect of sex.

To examine the effect of each grouping of covariates on the relationship between sex and our outcome we examined change in odds ratios (ORs) between our initial model and the model including each of the covariates. Relative change in OR was calculated by subtracting the initial OR from the OR in the second model, dividing by the initial OR, and then multiplying by 100.

### Ethics Approval

This study was approved by the Health Sciences Research Ethics Board of the University of Toronto (#29008) and the Monash University Human Research Ethics Committee (#CF09/3150-2009001727). This study analyzed data from an existing administrative database (the CRD) with de-identified information and did not involve direct interaction with participants. Therefore, informed consent was not applicable to our study. However, authorization for access to the dataset was obtained from ISCRR at Monash University.

## Results

### Description of Study Sample

A total of 1786 (63.1% male, 36.9% female) work-related TBI claims that included at least 10 days of wage replacement *or* surpassed the medical expenses threshold were lodged during the 2004–2012 period. Detailed characteristics of the study sample, by sex, are presented in Table 1. The average age of the sample was 40.6 years (SD = 13.5) and the majority of the claimants were working full-time (69.4%) and living in major cities of Australia (69.9%) at the time of their injury. Overall, 28.2% of claimants (33.7% of men, 18.8% of women) were hospitalized within seven days post-injury for a median of 1.0 day (IQR = 0–4.0); 1.0 day for men (IQR = 0–5.0) and 1.0 day for women (IQR = 0–2.0). Overall, 33.9% of claimants (36.5% of men, 29.6% of women) had at least 10 days of wage compensation during the first 30 days post-incapacity with a median number of days compensated of 15.0 days (IQR = 7.0–17.0); 16.0 days for men (IQR = 8.0–17.0) and 13.0 days for women (IQR = 5.0–16.0). Χ^2^ tests of independence determined that men and women differed in all categorical variables. Compared to women, men were more likely to be younger, hospitalized, compensated with wage replacement, working full-time, employed in lower skill-level jobs, and working for smaller employers. Women were more likely to live in major cities and in higher ranked socioeconomic advantage/disadvantage areas compared to men.

**Table 1:** Characteristics of Study Sample, By Sex.

	Total Sample	Men	Women
**Number of Claims, N (%)**	1786 (100.0)	1127 (63.1)	659 (36.9)
**Demographic Factors**			
***Age Group*: *n (%)***^a^			
15–24 years	252 (14.1)	154 (13.7)	98 (14.9)
25–34 years	412 (23.1)	267 (23.7)	145 (22.0)
35–44 years	379 (21.2)	252 (22.4)	127 (19.3)
45–54 years	427 (23.9)	249 (22.1)	178 (27.0)
55–64 years	271 (15.2)	169 (15.0)	102 (15.5)
65+ years	45 (2.5)	36 (3.2)	9 (1.4)
**Need Factors**			
***Length of First Hospital Stay*: *n (%)***^a^			
Not hospitalized	1282 (71.8)	747 (66.3)	535 (81.2)
0 days (day-patient)	159 (8.9)	108 (8.6)	51 (7.7)
1 day	141 (7.9)	107 (9.5)	34 (5.2)
2–3 days	71 (4.0)	49 (4.4)	22 (3.3)
4–7 days	45 (2.5)	39 (3.5)	6 (0.9)
8–14 days	^b^	^b^	^b^
15–30 days	^b^	^b^	^b^
31–90 days	47 (2.6)	41 (3.6)	6 (0.9)
***Days Compensated*: *n (%)***^a^			
No days	1180 (66.1)	716 (63.5)	464 (70.4)
1–5 days	132 (7.4)	79 (7.0)	53 (8.0)
6–10 days	70 (3.9)	38 (3.4)	32 (4.9)
11–15 days	123 (6.9)	76 (6.7)	47 (7.1)
16–20 days	245 (13.7)	189 (16.8)	56 (8.5)
21–31 days	36 (2.0)	29 (2.6)	7 (1.1)
**Work-Related Factors**			
***Employment Type*: *n (%)***^a^			
Full-time	1239 (69.4)	847 (75.2)	392 (59.9)
Part-time	228 (12.8)	73 (6.5)	155 (23.5)
Other	319 (17.9)	207 (18.4)	112 (17.0)
***Skill Level*: *n (%)***^a^			
1 (Highest)	385 (21.6)	161 (14.4)	224 (34.0)
2	146 (8.2)	83 (7.4)	63 (9.6)
3	356 (19.9)	281 (24.9)	75 (11.4)
4	478 (26.8)	324 (28.8)	154 (23.4)
5 (Lowest)	421 (23.5)	278 (24.6)	143 (21.7)
***Employer Remuneration Size*: *n (%)***^a^			
Small	566 (31.7)	440 (39.0)	126 (19.1)
Medium	621 (34.8)	393 (34.9)	228 (34.6)
Large	390 (21.8)	216 (19.2)	174 (26.4)
Government	209 (11.7)	78 (6.9)	131 (19.9)
**Geographic Factors**			
***Remoteness*: *n (%)***^a^			
Unknown	5 (0.3)	3 (0.3)	2 (0.3)
Major Cities	1248 (69.9)	752 (66.7)	496 (75.3)
Inner Regional	433 (24.2)	303 (26.9)	130 (19.7)
Outer Regional & Remote	100 (5.6)	69 (6.1)	31 (4.7)
***Socioeconomic Advantage/Disadvantage*: *n (%)***^a^			
Rank 1 (most disadvantaged)	148 (8.3)	102 (9.1)	46 (7.0)
Rank 2	119 (6.7)	90 (8.0)	29(4.4)
Rank 3	112 (6.3)	78 (6.9)	34 (5.2)
Rank 4	171 (9.6)	114 (10.1)	57 (8.7)
Rank 5	188 (10.6)	124 (11.0)	64 (9.7)
Rank 6	221 (12.4)	141 (12.5)	80 (12.2)
Rank 7	229 (12.9)	136 (12.1)	93 (14.1)
Rank 8	202 (11.3)	128 (11.4)	74 (11.3)
Rank 9	274 (15.4)	153 (13.6)	121 (18.4)
Rank 10 (most advantaged)	118 (6.6)	58 (5.2)	60 (9.1)

^a^ χ^2^ tests of independence between men and women significant at the p<0.05 level

^b^ Cell sizes <5

The majority of male claimants were working as machinery operators/drivers, technicians and trades workers, and labourers; and in the construction, transport/postal/warehousing, and manufacturing industries. The majority of females were working as professionals, community/personal service workers, and labourers; and in the education and training, healthcare and social assistance, and arts and recreation services industries. Detailed descriptive analyses by industry and occupation information are provided in Table 2.

**Table 2:** Occupation and Industry Characteristics, By Sex.

	Total Sample	Men	Women
**Total Number of Claims (N, %)**	1786 (100.0)	1127 (63.1)	659 (36.9)
**Occupation**^a^**: n (%)**			
Managers	151 (8.5)	108 (9.6)	43 (6.5)
Professionals	278 (15.6)	83 (7.4)	195 (29.6)
Technicians and trades workers	300 (16.8)	251 (22.3)	49 (7.4)
Community and personal service workers	252(14.1)	127 (11.3)	125 (19.0)
Clerical and administrative workers	79 (4.4)	26 (2.3)	53 (8.0)
Sales workers	93 (5.2)	35 (3.1)	58 (8.8)
Machinery operators/drivers	289 (16.2)	260 (23.1)	29 (4.4)
Labourers	344 (19.3)	237 (21.0)	107 (16.2)
**Industry**^b^**: n (%)**			
Agriculture, forestry and fishing	74 (4.1)	55 (4.9)	19 (2.9)
Mining	11 (0.6)	11 (1.0)	0
Manufacturing	199 (11.1)	163 (14.5)	36 (5.5)
Electricity, gas, water and waste services	16 (10.9)	^c^	^c^
Construction	175 (9.8)	^c^	^c^
Wholesale trade	108 (6.1)	79 (7.0)	29 (4.4)
Retail trade	115 (6.4)	64 (5.7)	51 (7.7)
Accommodation and food services	63 (3.5)	29 (2.6)	34 (5.2)
Transport, postal and warehousing	179 (10.0)	162 (14.4)	17 (2.6)
Information media and telecommunications	19 (1.1)	11 (57.9)	8 (1.2)
Financial and insurance services	8 (0.5)	^c^	^c^
Rental, hiring and real estate services	13 (0.7)	^c^	^c^
Professional, scientific and technical services	49 (2.7)	18 (1.6)	31 (4.7)
Administrative and support services	52 (2.9)	32 (2.8)	20 (3.0)
Public administration and safety	133 (7.5)	95 (8.4)	38 (5.8)
Education and training	221 (12.4)	61 (5.4)	160 (24.3)
Health care and social assistance	164 (9.2)	33 (2.9)	131 (19.8)
Arts and recreation services	118 (6.6)	63 (5.6)	55 (8.4)
Other	69 (3.9)	55 (4.9)	14 (2.1)

^a^ Occupation type was coded using the Australian and New Zealand Standard Classification of Occupations [33]

^b^ Industry type was coded using the Australian and New Zealand Standard Industrial Classification [34]

^c^ Cell sizes <5

### Description of Rehabilitation Services

In total, 55 971 health-related service visits were received by our sample in the first year of service utilization. Of these service visits, 4 329 (7.7%) were occupational therapy services, 12 139 (21.7%) were physiotherapy services, 2 121 (3.8%) were psychology services, and 807 (1.4%) were speech therapy services. Thus, out of all health-related services received by our sample, approximately one third (34.6%) were rehabilitation services (occupational therapy, physiotherapy, psychology, or speech therapy). A detailed description of these services by sex can be found in Table 3. Men received more individual, driving, exercise physiology, and hydrotherapy sessions compared to women, and women received more group and job/workplace/employment services compared to men.

**Table 3:** Number of Individual Rehabilitation Service Visits Received by Sample, by Service Type and Sex.

	Number of Individual Services Received					
	Total Sample		Men		Women	
	n	% within Rehab Type	n	% within Rehab Type	n	% within Rehab Type
**Occupational Therapy (N = 4,329)**						
Individual Session	1252	28.9	1134	34.9	118	10.9
Group Session	51	1.2	37	1.1	14	13
Unspecified	1299	30	1090	33.5	209	19.4
Job /Employment Services	1621	37.4	888	27.3	733	68
Driving Services	106	2.4	101	3.1	5	0.5
**Physiotherapy (N = 12,139)**						
Individual Session	1604	13.2	1444	19	160	3.5
Group Session	761	6.3	441	5.8	320	7.1
Unspecified	8279	68.2	4459	58.5	3820	84.5
Exercise Physiology	797	6.6	744	9.8	53	1.2
Hydrotherapy	693	5.7	523	6.9	170	3.8
Balance Physiotherapy	5	0.04	5	0.06	0	0
**Psychology (N = 2,121)**						
Individual Session	652	30.7	589	37.7	63	11.3
Group Session	33	1.6	33	2.1	0	0
Unspecified	1321	62.3	858	54.9	463	82.8
Neuropsychology Session	115	5.4	82	5.2	33	5.9
**Speech Therapy (N = 807)**						
Individual Session	602	74.6	553	76.5	49	58.3
Group Session	75	9.3	61	8.4	14	16.7
Unspecified	130	16.1	109	15.1	21	25

### Rehabilitation Service Utilization

User percentages and visit counts for each type of rehabilitation service are presented in Table 4. Out of all claims, 12.9% (14.1% of men, 10.9% of women) used occupational therapy with a median visit count of 12.0 (13.0 for men, 10.0 for women), 22.5% (20.8% of men, 25.5% of women) used physiotherapy with a median visit count of 14.0 (15.0 for men, 13.0 for women), 9.4% (10.0% of men, 8.4% of women) used psychology with a median visit count of 8 (9.5 for men, 7.0 for women), and 2.2% (3.0% of men, 0.9% of women) used speech therapy services with a median visit count of 12(11.0 for men, 17.0 for women) during the first 365 non-hospitalized days post-injury. Overall, 28.4% (27.2% of men, 30.7% of women) of claimants used at least one type of rehabilitation service.

**Table 4:** User Percentages and User Visit Counts, By Sex.

Rehabilitation Type	Total	Men	Women
**Occupational Therapy**			
Users (n, %)	230 (12.9)	158 (14.1)	72 (10.9)
User Visit Count (Med, IQR)^a^	12.0 (4.0–27.0)	13.0 (4.0–27.0)	10.0 (4.0–24.5)
**Physiotherapy**			
Users (n, %)	402 (22.5)	234 (20.8)	168 (25.5)
User Visit Count (Med, IQR)^a^	14.0(5.0–45.0)	15.0 (5.0–49.5)	13.0 (6.0–39.0)
**Psychology**			
Users (n, %)	168 (9.4)	113 (10.0)	55 (8.4)
User Visit Count (Med, IQR)^a^	8.0 (3.0–17.0)	9.5 (4.0–17.0)	7.0 (3.0–17.0)
**Speech Therapy**			
Users (n, %)	40 (2.2)	34 (3.0)	6 (0.9)
User Visit Count (Med, IQR)^a^	12.0 (5.0–23.0)	11.0 (5.0–11.0)	17.0 (5.0–18.0)
**≥1 Type of Rehab**			
Users (n, %)	509 (28.5)	305 (27.1)	204 (30.9)

^a^ Visit counts calculated for users of each type of rehabilitation only

Results of the ZINB regression models are presented in Table 5. A model was not conducted for speech therapy due to the small number of users (n = 40). Men and women were equally likely to use occupational therapy and psychology services and had equal numbers of occupational therapy and psychology visits. No sex differences were evident for occupational therapy and psychology services either before or after adjusting for need, work-related, and geographic factors. Utilization of physiotherapy services differed for men and women with men being significantly less likely than women to use physiotherapy even after adjusting for work-related, geographic, and need factors. However, though less likely to be a physiotherapy user, men who did use physiotherapy services did not differ from women in the number of times they received the service over the first year.

**Table 5:** ZINB Estimates for Use and Number of Occupational Therapy, Physiotherapy, and Psychology Visits (Men Relative to Women).

	Logistic Regression Component (Predicting Use vs. Non-Use)			Negative Binomial Component (Predicting Visit Count)		
	Estimate	CI 95%	p-value*	Estimate	CI 95%	p-value*
**Occupational Therapy**						
***Model*: *Sex***						
OR	1.32	0.97, 1.80	0.0810	1.15	0.78, 1.68	0.4755
***Model*: *Work-Related Factors***						
OR	0.99	0.70, 1.40	0.9574	1.28	0.83, 1.98	0.2695
Relative Change in OR	-25.0%			+11.3%		
***Model*: *Geographic Factors***						
OR	1.30	0.95, 1.78	0.0988	1.23	0.84, 1.79	0.2913
Relative Change in OR	-1.52%			+7.0%		
***Model*: *Need Factors***						
OR	0.78	0.52, 1.16	0.2154	0.71	0.49, 1.02	0.0616
Relative Change in OR	-40.9%			-38.3%		
***Model*: *All Factors***						
OR	0.68	0.43, 1.06	0.0888	0.93	0.63, 1.38	0.7275
Relative Change in OR	-48.4%			-19.1%		
**Physiotherapy**						
***Model*: *Sex***						
OR	0.74	0.58, 0.95	0.0162*	1.16	0.88, 1.52	0.2984
***Model*: *Work-Related Factors***						
OR	0.69	0.53, 0.91	0.0081*	1.07	0.78, 1.46	0.6823
Relative Change in OR	-6.76%			-7.76%		
***Model*: *Geographic Factors***						
OR	0.74	0.58, 0.95	0.0198*	1.20	0.90, 1.59	0.2194
Relative Change in OR	None			+3.45%		
***Model*: *Need Factors***						
OR	0.54	0.42, 0.71	< .0001*	0.79	0.60, 1.05	0.1083
Relative Change in OR	-27.0%			-31.90%		
***Model*: *All Factors***						
OR	0.56	0.41, 0.77	0.0003*	0.85	0.62, 1.18	0.3328
Relative Change in OR	-24.3%			-26.7%		
**Psychology**						
***Model*: *Sex***						
OR	2.04	0.83, 1.69	0.3435	1.39	0.94, 2.06	0.0977
***Model*: *Work-Related Factors***						
OR	1.19	0.81, 1.75	0.3871	1.35	0.86, 2.13	0.1945
Relative Change in OR	-41.7%			-2.9%		
***Model*: *Geographic Factors***						
OR	1.19	0.83, 1.71	0.3417	1.50	0.99, 2.24	0.0585
Relative Change in OR	-41.7%			+7.9%		
***Model*: *Need Factors***						
OR	0.76	0.50, 1.16	0.2086	1.15	0.75, 1.77	0.5112
Relative Change in OR	-62.7%			-17.3%		
***Model*: *All Factors***						
OR	0.91	0.57, 1.45	0.6779	1.35	0.83, 2.20	0.2248
Relative Change in OR	-55.4%			-2.9%		

All models are age-adjusted and year-adjusted

CI, confidence interval; OR, odds ratio

* *p*-value statistically significant at the 0.05 level

Comparing each of the three types of factors, need factors consistently caused a large change in the logistic (user/non-user) and negative binomial (visit count) estimates compared to work-related and geographic factors. Adjusting for need factors consistently shifted the estimates for men in a negative direction (i.e., showed men to be less likely to use rehabilitation and have lower visit counts compared to women). This was due to men being more likely to have need factors that were associated with greater service use (both in general and number of services) compared to women. Adjusting for work-related and geographic factors caused little change in the logistic and negative binomial estimates for all three types of rehabilitation, with the exception of occupational therapy use where adjusting for work-related factors significantly shifted the estimate for men in the negative direction.

## Discussion

Using a population-based sample, this study is one of the first to examine the characteristics of the utilization of occupational therapy, physiotherapy, psychology, and speech therapy services following a work-related TBI, as well as differences in utilization between men and women.

Our results are consistent with previous TBI studies who report that physiotherapy services are used by the greatest number of people with TBI compared to other types of rehabilitation, such as occupational therapy, psychology, and speech therapy. [21–23, 25] In fact, Prang et al. [21] found physiotherapy to be used by the greatest number of people, following by occupational therapy, psychology, and speech therapy, which is consistent with the order of highest to lowest use in our study. However, the absolute proportion of TBI cases to use each type of rehabilitation service varied significantly across studies [21–23,25] which is likely due to differences in data sources and therefore study populations and injury severities (e.g., hospitalizations, transport-related injuries, etc.). Another similarity to past literature is that in our study, need factors (length of first hospital stay and number of days compensated) caused the greatest change in model estimates which is consistent to Willemse-van Son et al.’s [22] finding that need factors (specifically, restrictions in participation and co-morbidities) explained most of the variance in rehabilitation use.

Our results regarding sex differences in rehabilitation utilization did not completely align with our hypotheses. We did expect to see utilization differences between men and women and did so in the utilization of physiotherapy, but did not find sex differences in the utilization of occupational therapy and psychology. In addition, although we did expect to see sex-differences in physiotherapy use, the direction of this difference is surprising. The goal of rehabilitation within this workers’ compensation system is to return to work. Since men in our sample work in more physically demanding jobs compared to women, one may expect regaining physical function to be a higher priority for rehabilitation for men, and therefore would expect men to be more likely than women to use physiotherapy and at a higher intensity. However, in our sample women were more likely than men to use physiotherapy. With that being said, the difference in the proportion of men and women who used physiotherapy was just 4.8%, so whether or not this difference is clinically significant remains open to interpretation. However, it should be noted that this difference of 4.8% does not take into account the greater need of male work-related TBI claimants, as measured by days of compensation in the first 30 days and length of hospitalization.

Our finding that the use of occupational therapy and psychology services did not differ between sexes is consistent with a previous study that examined service utilization of persons with a TBI at four regional TBI centers in New York State [23]. This study reported the utilization of physiotherapy, occupational therapy, speech therapy, therapeutic recreation, and psychology services were similar for men and women. However, this is inconsistent with our other finding that women were more likely to use physiotherapy compared to men. In addition, our finding that men and women did not differ in the number of times they used each type of service is inconsistent with Kristman et al.’s [25] study that found women had a higher rate of health service utilization (number of services per 1000 claimants) compared to men following a mild work-related TBI. However, Kristman et al. [25] examined all healthcare services together and did not examine rehabilitation separately. Overall, it is difficult to compare our findings to those of past literature because our study is the first to examine sex differences in the use of specific types of rehabilitation services in a work-related TBI population. More studies like ours need to be conducted in order to make better comparisons and more accurate conclusions.

Some limitations are worth noting. First, some of the study sample may have used services that they have not claimed under the workers’ compensation system but instead under Medicare or a private health insurer. [23] Second, the data source lacks detailed information about (i) the severity of the TBI (e.g., Glasgow Coma Score) and (ii) the short term consequences as a result of the injury (e.g., cognitive impairment measured by cognitive tests conducted shortly after the injury, physical impairment due to brain damage or secondary injuries sustained simultaneously, etc.). Without more detailed information about the nature of the TBI sustained and the resulting cognitive, physical, and functional consequences, it was not possible to determine whether the rehabilitation services provided were appropriate for each claimant’s need. As a result we cannot comment on whether women’s increase prevalence of physiotherapy use (compared to men) is the result of a healthcare inequity, or the result of detailed differences in the TBI injuries sustained by women compared to men.

Some strengths of our study include our objective measures of service use (e.g., service payment information), which is unique as many existing studies rely on self-reported healthcare use, which can be a problem particularly for TBI patients (e.g., memory deficits[25]). Also, many studies using hospital data only include hospitalized cases and exclude milder injuries that were not hospitalized, while our study includes both hospitalized and non-hospitalized injuries and thus captures a wider range of TBI severity. This allows us to develop a more comprehensive picture of rehabilitation service utilization by this population and increase generalizability to other work-related TBI populations. Our findings may also be generalizable to other jurisdictions in Australia and in other countries where similar compensation systems operate (e.g., Canada). In fact, Canadian and Australian compensation systems are quite similar—future research should replicate our study using Canadian workers’ compensation data in order to make comparisons between both countries.

In conclusion, this study is one of the first comprehensive sex-based examinations of the utilization of occupational therapy, physiotherapy, psychology, and speech therapy services following a work-related TBI in Victoria, Australia. While women were more likely to use physiotherapy compared to men, both were equally likely to use occupational therapy and psychology services, and the intensity of rehabilitation use (i.e., number of visits) did not differ between sexes for each type of service. Future research should focus on (1) other factors associated with rehabilitation service use among the work-related TBI population (e.g., age and work-related factors), (2) costs associated with rehabilitation service utilization, and (3) whether different patterns of rehabilitation utilization result in different health and return to work outcomes.
